# Synthetic melanin bound to subunit vaccine antigens significantly enhances CD8^+^ T-cell responses

**DOI:** 10.1371/journal.pone.0181403

**Published:** 2017-07-17

**Authors:** Antoine F. Carpentier, Frédéric Geinguenaud, Thi Tran, Floraly Sejalon, Antoine Martin, Laurence Motte, Eric Tartour, Claire Banissi

**Affiliations:** 1 Université Paris 13, Sorbonne Paris Cité, Bobigny, France; 2 Laboratoire de Recherches Biochirurgicales, Université Paris Descartes, Hôpital Européen Georges Pompidou, Paris, France; 3 Hôpital Avicenne, Assistance Publique-Hôpitaux de Paris (AP-HP), Bobigny, France; 4 Laboratoire CSPBAT, CNRS UMR 7244 UFR SMBH, Université Paris 13, Sorbonne Paris Cité, Bobigny, France; 5 INSERM U970, Université Paris Descartes, Sorbonne Paris-Cité. Equipe Labellisée Ligue Contre le Cancer, Hôpital Européen Georges Pompidou, Paris, France; 6 Service d’Anatomie et Cytologie Pathologiques, Hôpital Avicenne, Assistance Publique-Hôpitaux de Paris (AP-HP), INSERM U978, Université Paris 13, Sorbonne Paris Cité, Bobigny, France; 7 INSERM U1148, Laboratory for Vascular Translational Science, UFR SMBH, Université Paris 13, Sorbonne Paris Cité, Bobigny, France; 8 Assistance Publique-Hôpitaux de Paris (AP-HP), Hôpital Européen Georges Pompidou, Service d'Immunologie biologique, Hôpital Européen Georges Pompidou, Paris, France; Monash University, AUSTRALIA

## Abstract

Cytotoxic T-lymphocytes (CTLs) play a key role in immunity against cancer; however, the induction of CTL responses with currently available vaccines remains difficult. Because several reports have suggested that pigmentation and immunity might be functionally linked, we investigated whether melanin can act as an adjuvant in vaccines. Short synthetic peptides (8–35 amino acids long) containing T-cell epitopes were mixed with a solution of L-Dopa, a precursor of melanin. The mixture was then oxidized to generate nanoparticles of melanin-bound peptides. Immunization with melanin-bound peptides efficiently triggered CTL responses in mice, even against self-antigens and at a very low dose of peptides (microgram range). Immunization against a tumor antigen inhibited the growth of established tumors in mice, an effect that was abrogated by the depletion of CD8^+^ lymphocytes. These results demonstrate the efficacy of melanin as a vaccine adjuvant.

## Introduction

The recent developments in the ability to identify genetic mutations that can be recognized by the immune system (neo-epitopes), along with the success of immune checkpoint inhibitors, have renewed the interest for cancer vaccines [1,2,3]. Antibody responses, commonly induced with modern vaccines; are not helpful where intra-cellular antigens are involved, which is usually the case in cancer. Immunity in cancer is mainly mediated by cytotoxic T- lymphocytes (CTLs) [4,5]. CTLs recognize short peptidic epitopes (eight to ten amino acid residues) that result mainly from degraded intracellular proteins, and that are displayed by major histocompatibility complex (MHC) class I molecules on the surfaces of target cells and/or antigen presenting cells (APCs). However, CTL responses are much more difficult to trigger in humans than antibody responses [4], as the induction of CTL responses requires the extracellular antigen (usually presented by MHC class II molecules) to be redirected to the MHC class I pathway in a process called cross-presentation [6]. To achieve cross-presentation, and subsequently the induction of CTL responses, several formulations have been developed, including viral vectors, water-in-oil emulsions, dendritic cell purifications, DNA vaccines, and combinations with immune adjuvants such as Toll-like receptors (TLRs) or toxins [4, 5, 7–11]. However, these approaches face barriers to clinical development, such as poor stability, high manufacturing costs, and inadequate immune responses in humans [12,13]. Another limitation is seen with subunit vaccines which use a small part of an antigen. Indeed, peptides are typically poorly immunogenic, and both adjuvants and high antigenic doses are therefore usually needed to elicit an effective immune response [12,13].

Melanin, synthetized within the melanosomes of epidermal melanocytes, is widely found in mammalian skin and plays a major role in the protection of skin cells against mutagenic ultraviolet (UV) light rays [14]. Interestingly, melanocytes exhibit phagocytic functions, and phagosomes are transported from the cell surface to the melanosomes, supporting the idea that melanosomes, that contain many lysosomal enzymes, are part of the lysosomal degradation pathway [15–17]. Further studies have shown that melanocytes may contribute to the phagocytosis of invading pathogens and can act as antigen presenting cells [15,18]. We therefore investigated whether melanin itself could act as an adjuvant for immune responses against specific antigens.

Here, we show that the attachment of synthetic melanin to small peptidic epitopes can be used in vaccine formulations to efficiently trigger immune responses. Moreover, the addition of synthetic melanin to the vaccine formulation enables a dramatic reduction in antigen dose compared with the doses required for other conventional vaccine approaches.

## Results

### Preparation and characterization of synthetic melanin and peptide vaccine formulations

**Fig 1:**
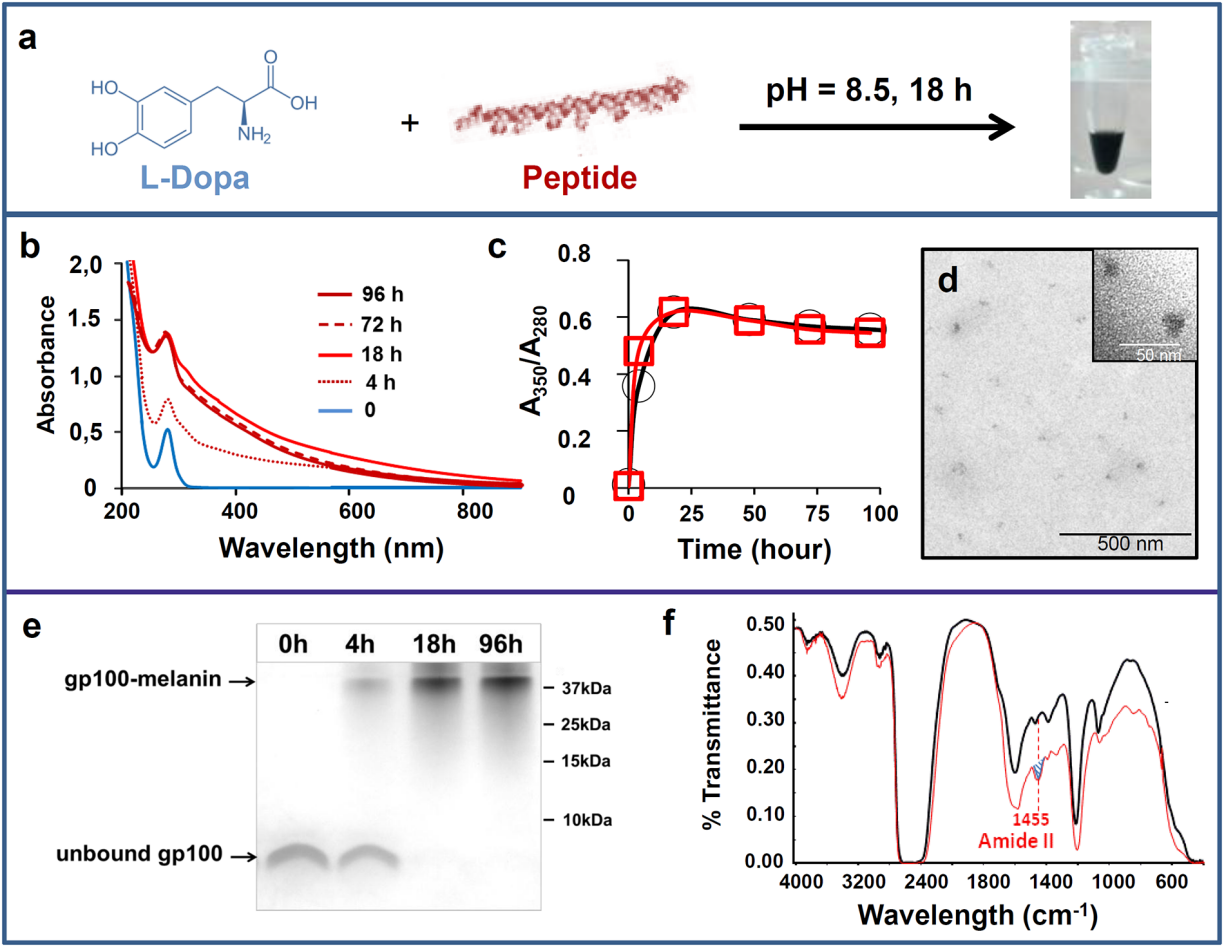
Preparation and characterization of synthetic melanin and peptide vaccine formulations. (a) Schematic of the chemical process for obtaining water-soluble peptide-melanin complexes. Evolution over time of b) the UV-visible spectrum during gp100-melanin synthesis and c) the absorbance ratio at 350 and 280 nm (A350/A280) for melanin (black squares) and for gp100-melanin (red circles). (d): TEM image of gp100-melanin after 18 h of incubation time (inset: high magnification). (e) SDS-PAGE showing the migration of the unbound gp100 peptide within the resolving gel after different incubation times in the presence of oxidizing L-Dopa leading to gp100-melanin formation (following electrophoresis, the gels were stained with Coomassie Blue). (f) FTIR spectra in deuterated solution for melanin (black line) and gp100-melanin (red line); the amide II' band at 1455 cm-1 (blue hatching) is characteristic of peptides.

The oxidation of L-Dopa in alkaline conditions is an established process for obtaining synthetic melanin [19]. Thus, to prepare the vaccine formulations, a solution of L-Dopa was mixed with a solution of peptide corresponding to the human gp100 epitope [20] (weight ratio L-Dopa: epitope of 1:4), and the mixture was then oxidized at pH 8.5 in aerated conditions (Fig 1a). Under these conditions, the colorless L-Dopa solution turned black, a process that we monitored using UV spectroscopy (Fig 1b). The kinetics of L-Dopa oxidation, as assessed by the 350/280 nm ratio, were similar with or without peptide (Fig 1c). When filtered through a 10-kDa filter, a black material was retained in the upper chamber, and could be easily resuspended in a saline solution. Transmission electronic microscopy (TEM) images showed nanoparticles with sizes mainly between 10 and 20 nm (Fig 1d). When the reaction was monitored via SDS-PAGE, the free peptide disappeared within 18 h (Fig 1e). This suggests that melanin bound to gp100 (forming gp100-melanin). The binding of the peptide to gp100 was confirmed by FTIR spectroscopy. The amide II' band at 1455 cm-1, which is characteristic of peptides [21] was observed with the gp100-melanin spectrum but not with the control melanin spectrum (Fig 1f).

### Distribution of melanin in draining lymph nodes

**Fig 2:**
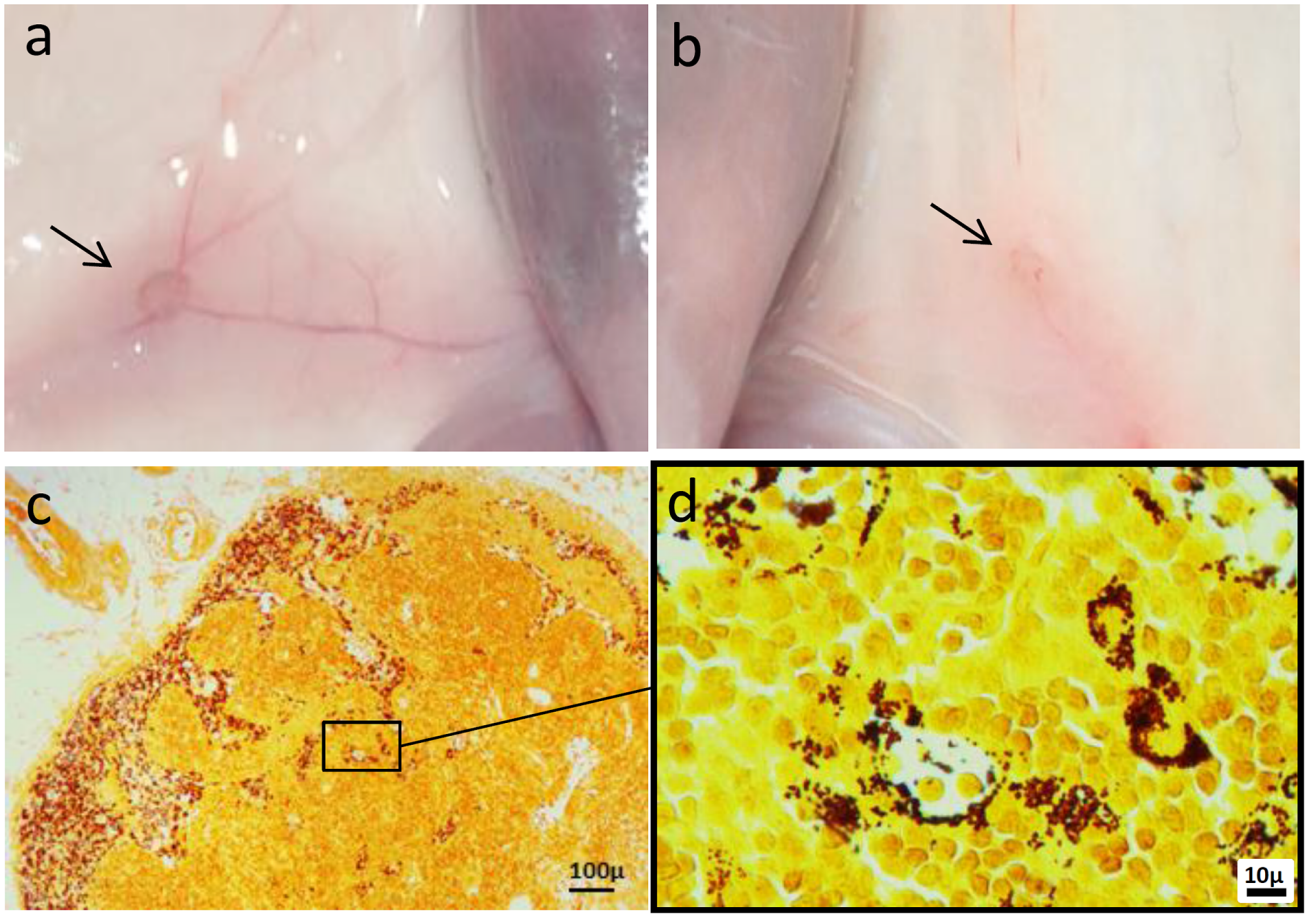
Distribution of melanin in draining lymph nodes. Macroscopic aspect of the draining inguinal lymph nodes (arrows) of BALB/c mice 2 days after injections with gp100-melanin (a) or saline (b). Fontana-Masson staining of a draining lymph node 2 days after injection with gp100-melanin, showing melanin-laden macrophages in the sinuses (c) and in the paracortical area (d).

The induction of antigen-specific immunity relies on the direct interaction of DCs with naive T-cells that occur in the T-cell zone of lymph nodes. To assess the distribution of the vaccine formulations *in vivo*, mice were injected subcutaneously with [gp100-melanin + CpG-28] or saline, and sacrificed on days 2 or 7 (n = 3/group). To avoid any bias caused by natural melanin, these experiments were carried out in BALB/c mice, which are naturally devoid of melanin. Black pigmentation of the draining inguinal lymph nodes was macroscopically visible on day 2 post-injection in gp100-melanin-injected animals (Fig 2a & 2b). Fontana-Masson staining confirmed numerous melanin-laden macrophages in the sinuses (Fig 2c) and, to a lesser extent, in the paracortical areas, which is a T-cell zone (Fig 2d). The pattern of melanin distribution was similar at days 2 and 7 post-injection, and in mice injected with gp100-melanin (without CpG-28). No melanin was observed in mice that received saline only. These results show that the vaccine formulation effectively reached the draining lymph nodes in vivo.

### Immunization with low-dose gp100-melanin triggers CTL responses

**Fig 3:**
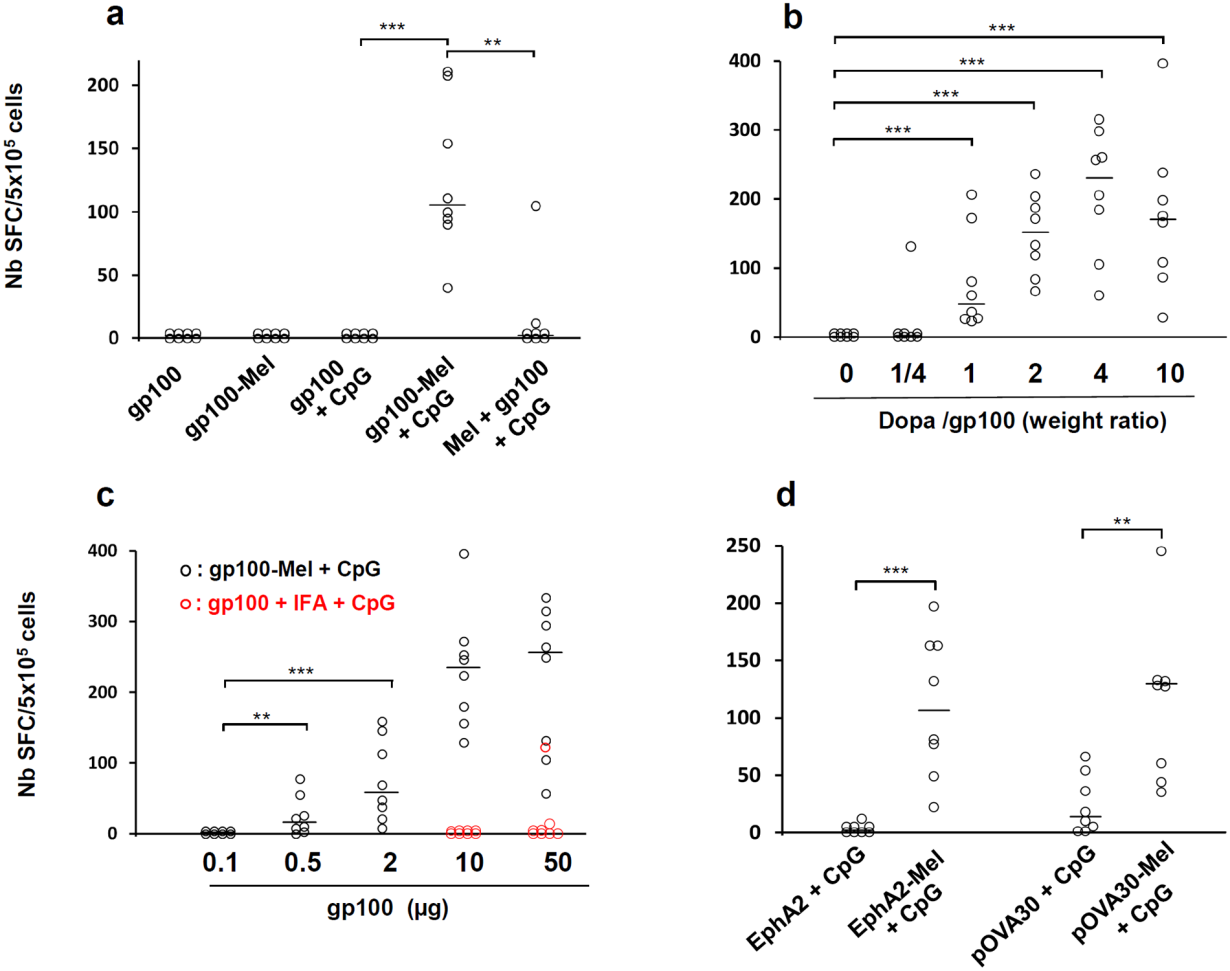
CTL response after subcutaneous immunizations in C57BL/6 mice. (a) Mice were immunized with gp100, gp100 + CpG, gp100-melanin (gp100-Mel), gp100-melanin + CpG (gp100-Mel + CpG), or previously synthesized melanin mixed with gp100 and CpG (Mel + gp100 + CpG). (b) Mice were immunized with gp100-melanin, synthesized with 10 μg gp100 and L-Dopa at various weight ratios. (c) Mice were immunized with gp100-melanin synthesized with various amounts of the gp100 epitope and L-Dopa at a weight ratio of 1:4 (gp100-Mel + CpG, black open circles). Some mice were immunized with 10 or 50 μg of gp100 in combination with incomplete Freund’s adjuvant and 50 μg of CpG (gp100 + IFA + CpG; red open circles). (d) Mice were immunized with EphA2 + CpG, EphA2-melanin + CpG [EphA2-Mel + CpG], pOVA30 + CpG, or pOVA30-melanin + CpG [pOVA30-Mel + CpG]. In a), b) and d), mice were immunized on days 0 and 7 and sacrificed on day 14. In c), mice were immunized on day 0 and sacrificed on day 8. Each point represents an individual mouse (n = 8 mice/group with pooled data from 2 different experiments of 4 mice each). SFC: spot-forming cells; Bars = median. ** p < 0.01; ***p<0.001 (Mann–Whitney test).

We then assessed the ability of gp100-melanin to trigger an immune response in mice. Free gp100 peptide and gp100-melanin and were used as vaccine preparations alone or mixed with the TLR9 agonist CpG-28 (CpG) [9]. When combined with CpG, gp100-melanin, but not gp100, induced a significant number of IFNγ-secreting lymphocytes (p<0.001) (Fig 3a). Similar data were obtained when a TLR3 agonist was used (S1 Fig). If the gp100 epitope was added in the vaccine formulation after L-Dopa had been oxidized instead of before, no significant CTL response was observed (p<0.01) (Fig 3a). The minimal weight ratio of L-Dopa:epitope required to induce significant immunity was 1:1, with the best response observed at a ratio of 4:1 (Fig 3b). The minimal dose of the gp100 epitope required to induce CTLs was 0.5 μg (p<0.01, when compared to the lowest concentration tested) (Fig 3c). At both 10 and 50μg of the gp100 epitope, our vaccine formulation compared favorably (p<0.01) with the combination of incomplete Freund’s adjuvant (IFA) and a TLR9 agonist, a combination that is commonly used to trigger CTL responses [22,23] (Fig 3c). After two 2 initial immunizations with gp100-melanin + CpG, the specific CTL response progressively diminished over a period of months and was still seen after 4 months (S2 Fig).

### Immunization against other MHC class I epitopes also triggers CTL responses

We then checked that the efficacy of our vaccine combining synthetic melanin and CpG was not limited to the gp100 epitope and can be generalized to other peptides containing MHC class I epitopes. We investigated the ability of this formulation to trigger CTL responses with a self-epitope derived from the murine Ephrin-A2 protein (EphA2) and with a long synthetic peptide (30 amino acids) containing the classic ovalbumin SIINFEKL epitope (pOVA30). The peptides were incubated for 18 h with L-Dopa to generate EphA2-melanin and pOVA30-melanin and then combined with CpG for immunizations. These epitopes alone were not immunogenic or were poorly immunogenic, whereas immunization with either EphA2-melanin or pOVA30-melanin induced the production of a significant number of IFNγ-secreting lymphocytes (p<0.001 and p<0.01 for EphA2 and pOVA30, respectively) (Fig 3d). Taken together with the results described above, this suggests combination with melanin successfully increased the CTL-based immune response to vaccination against various peptidic epitopes.

### Immunization with the melanin-adjuvant vaccine formulation induces effector memory CD8^+^ T-cells

**Fig 4:**
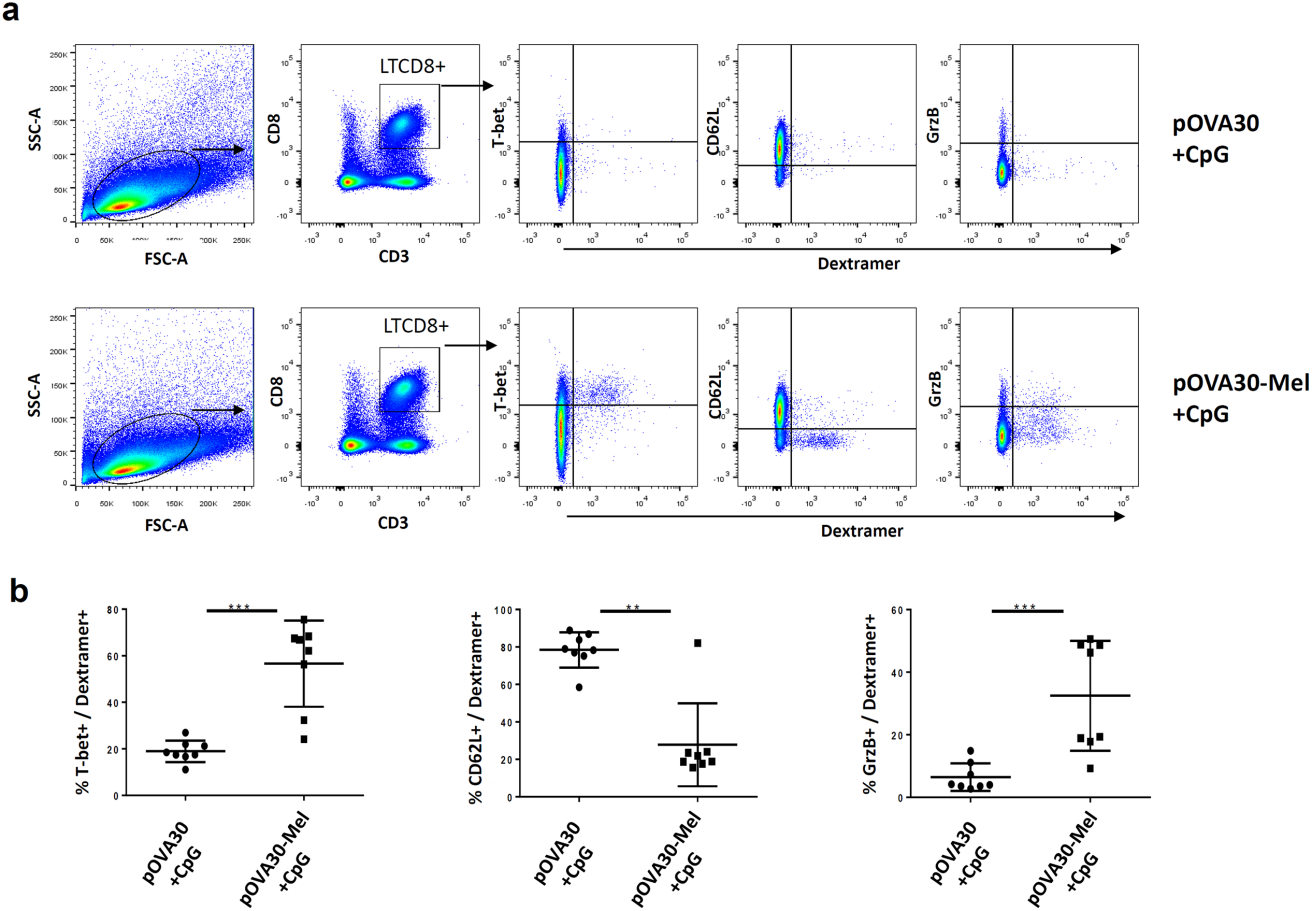
Phenotype of SIINFELKL-specific T-cells in mice immunized on days 0 and 14 with [pOVA30 + CpG] or [pOVA30-melanin + CpG]. (a) gating strategy and representative results (in one mouse) for T-Bet, CD62L and granzyme expression. (b) mean ± SD expression of T-bet, CD62L and granzyme within the CD8^+^dextramer^+^ population (n = 8 mice/group, with pooled data from two different experiments of 4 mice each). ** p<0,01; ***p<0,001.

To characterize the CTL phenotype, mice were immunized subcutaneously with either [pOVA30 + CpG] or [pOVA30-melanin + CpG] on days 0 and 14 and sacrificed on day 21. The percentage of SIINFEKL-specific T cells (dextramer^+^ T-cells) within the CD8^+^ lymphocyte population was significantly enhanced in mice given [pOVA30-melanin + CpG] compared with mice given [pOVA30 + CpG] (mean ± SD: 1.42 ± 0.13% vs 0.1 ± 0.04%, respectively; p = 0.05) (data not shown). Analysis of these dextramer^+^ CD8^+^ cells using multiparametric flow cytometry showed a phenotype of effector memory CD8^+^ T-cells, with low CD62L expression and high T-bet and granzyme expression in the [pOVA30-melanin + CpG] group (Fig 4).

### Subcutaneous injections of pOVA30-melanin protect against established syngenic tumors

**Fig 5:**
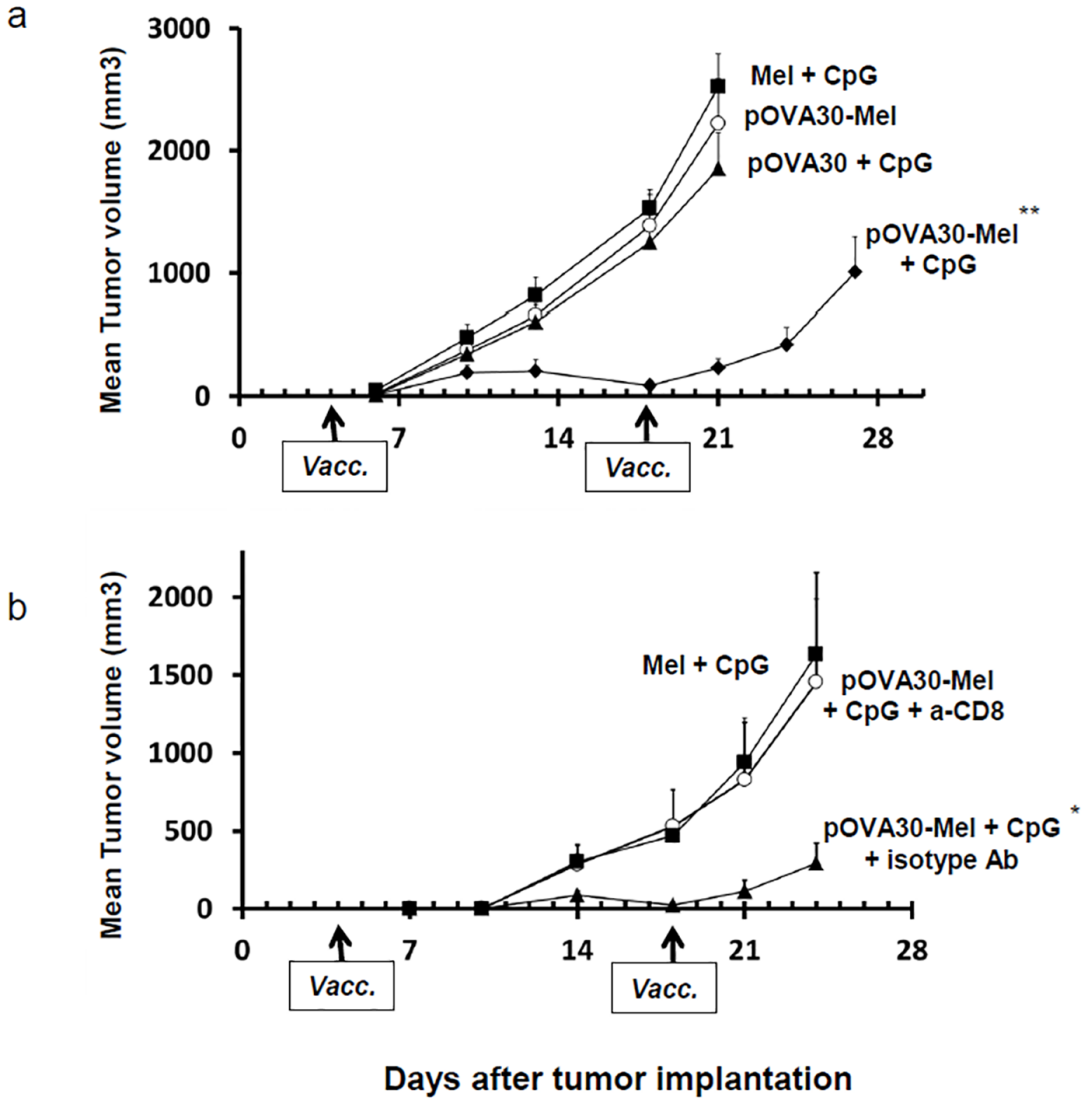
Anti-tumor effect of immunizations in C57BL/6 mice bearing subcutaneous tumors. (a) Mice were injected with E.G7-OVA cells on day 0 and then immunized on days 4 and 18 (arrows) with [pOVA30-melanin + CpG], [melanin + CpG], [pOVA30 + CpG], or [pOVA30-melanin] (n = 10 mice/group with pooled data from two different experiments). (b) Effect of the in vivo CD8 depletion in C57BL/6 mice bearing subcutaneous E.G7-OVA tumors and immunized on days 4 and 18 (arrows) with [melanin + CpG] (n = 4 mice) or [pOVA30-melanin + CpG] (n = 8 mice). The latter group received either anti-CD8 or an isotype-matched control mAb. Tumor growth was assessed twice a week by measuring the size of tumors with calipers. Results are expressed as the mean ± SEM of tumor volumes. * p < 0.05; ** p < 0.001, when compared with control groups.

We next investigated whether these CD8^+^ T-cells were functional in vivo. Ovalbumin-transfected cells (E.G7-OVA) were injected subcutaneously into C57BL/6 mice, and the mice were immunized on days 4 and 18 with [melanin + CpG-28], [pOVA30-melanin + CpG-28], [pOVA30 + CpG-28], or [pOVA30-melanin]. All of the mice developed measurable tumors. A significant decrease in the tumor growth compared with that in the control groups was observed only after immunization with [pOVA30-melanin + CpG-28] (p<0.001) (Fig 5a). Complete tumor regression occurred in 2/10 mice. Depletion of CD8^+^ cells by monoclonal antibodies abrogated this anti-tumor effect (Fig 5b). These results suggest that the vaccine elicited an immune response that allowed a tumor growth inhibition, which was mediated by CD8^+^ T-cells.

### Immunization with melanin formulation without any TLR agonist

**Fig 6:**
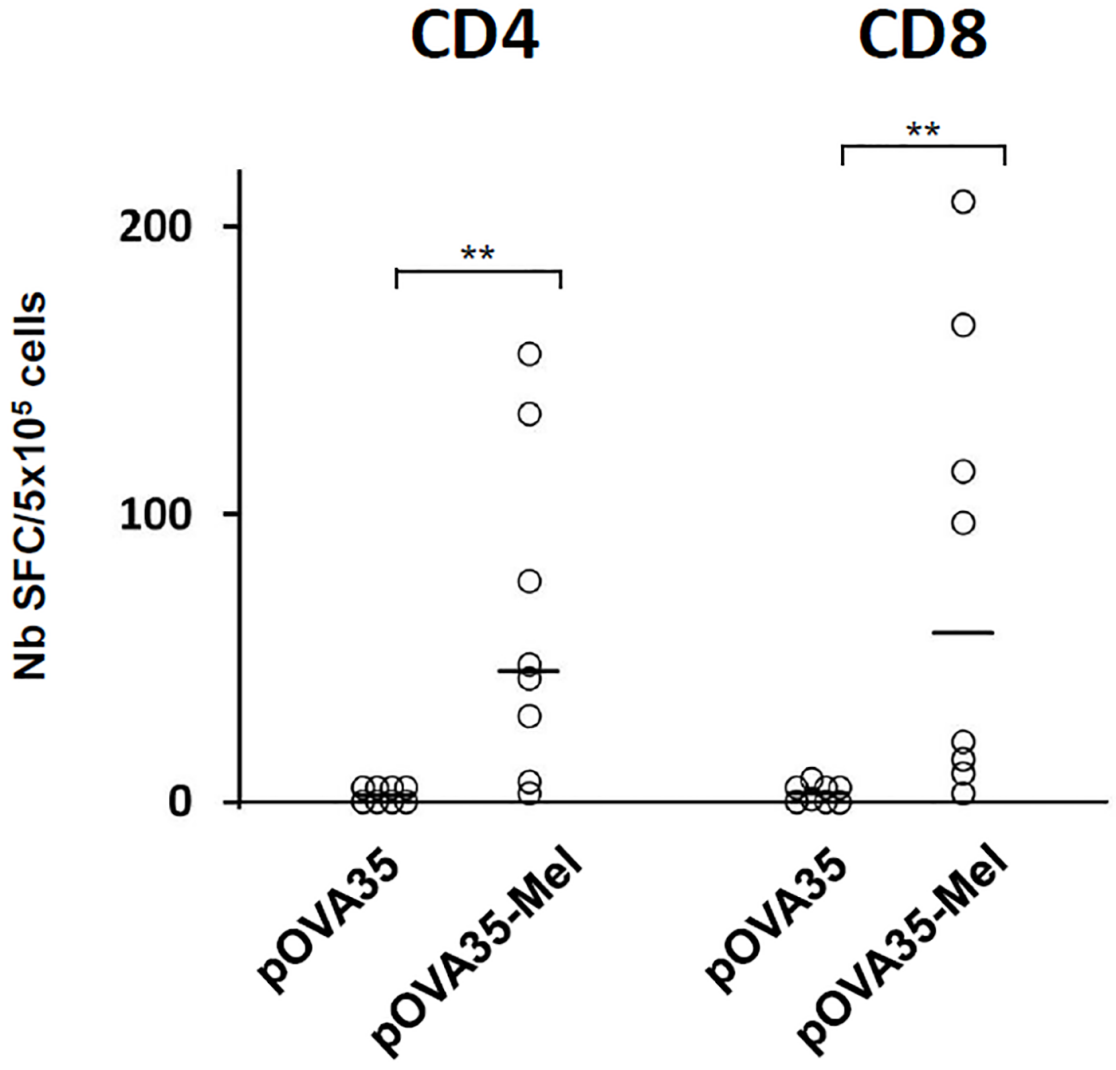
T-cell response after subcutaneous immunizations in C57BL/6 mice. Mice were immunized with pOVA35 or pOVA35-melanin (pOVA35-Mel), on days 0 and 14 and sacrificed on day 21. Splenocytes were re-stimulated in vitro either with a) the MHC class II epitope (CD4) or b) the MHC class I-epitope (CD8) (non conjugated to melanin). The numbers of IFNg-SFCs (Spot forming cells) were measured. Each point represents an individual mouse (n = 8 mice/group with pooled data from 2 different experiments of 4 mice each). Bars = median. ** p < 0.01 (Mann–Whitney test).

We finally assessed the ability of our formulation to trigger an immune response without any TLR agonist. The synthetic peptide pOVA35, that contains both a MHC class II (ISQAVHAAHAEINEAGC) and a MHC class I (SIINFEKL) ovalbumine epitopes, was incubated for 18 h with L-Dopa to generate pOVA35-melanin. Mice were immunized subcutaneously with either free pOVA35 or pOVA35-melanin on days 0 and 14 (without CpG), and the immune response was assessed on day 21. The pOVA35 peptide alone was not immunogenic, whereas immunization with pOVA35-melanin induced the production of a significant number of IFNγ-secreting lymphocytes against the CD4 epitope (p<0.01) (Fig 6). The presence of the MHC-class II epitope allowed the mice immunized with pOVA35-melanin, but not those immunized with pOVA35 only, to mount a significant CTL response against the MHC-class I epitope (p<0.01) (Fig 6).

## Discussion

Protection against UV radiation and radical scavenging are the most acknowledged functions of melanin in mammals [14]. Here, we showed that melanin, when synthesized in the presence of antigens, may play a role in antigen-specific immunity. Indeed, epitope-containing peptides associated with synthetic melanin triggered specific immune responses, with effector memory CD8+ lymphocytes expressing granzyme. These immune responses were functional, because immunization against tumor antigens elicited an anti-tumor effect *in vivo*. Furthermore, induction of CD8+ lymphocytes with a short MHC class I epitope can be easily obtained either by embedding a MHC class II epitope within the peptides, or by adding a TLR agonist to the vaccine formulation, thus providing the “second signal” for cross-presentation [4,24].

The mechanism underlying the efficacy of our approach most likely involves an antigen carrier effect provided by melanin. Indeed, no immune response was observed if peptides were added after L-Dopa had been oxidized. This observation does not support an immunostimulatory effect of melanin itself on immune cells but instead indicates that a close association between the peptides and synthetic melanin is required. Cross-linking reactivity is a well-known property of catechol moieties and L-DOPA after oxidation [25,26], and the association between melanin and our peptides was indeed documented by FITR spectroscopy. After subcutaneous injection of our peptide-melanin nanoparticles, melanin was found in the draining lymph nodes. Such a migration within lymph nodes, in which T-lymphocytes are primed, is commonly observed with nanoparticles ≤200nm [27]. In humans, melanin deposits are commonly described in cases of dermatopathic lymphadenopathy, a lymph node pathology secondary to skin disease [28]. Interestingly, melanin deposits in our model were mainly seen in the sinuses and in the paracortex, which is a T-cell zone, and were still detected within lymph nodes 7 days after injection. It might be hypothesized that this slow clearance of melanin contributes to the efficacy of our vaccines by enabling a slow antigen release.

Cross-presentation of antigens by nanoparticulate formulations is a well-described process in immunology [29]. As synthetic melanin aggregates into nanoparticles [19], its ability to enhance immune responses to combined to antigens thus appears logical. Whether this phenomenon spontaneously occurs with natural melanin in living animals to protect against infections is unclear. In invertebrates, melanin plays a role in immune defense by encapsulating invading pathogens, a process known as melanization. However, such a process does not trigger specific immunity and does not appear to occur in vertebrates [30]. Yet, several facts support the idea that immunity and pigmentation are functionally linked in mammals. Numerous studies have suggested that darker-skinned individuals may be less susceptible to bacterial and fungal skin diseases [31]. In addition, in mice with melanocytosis, melanin granules in the skin are continuously captured and transported to regional lymph nodes by Langerhans cells [32,33]. Finally, normal melanocytes display antigen-presenting functions [15,18]. However, the role of melanin itself in these immune processes has never been addressed. Our results showing that melanin can act as an antigen carrier shed new light on the putative mechanism involved in the above described observations.

The vaccine formulation presented in this study has three major advantages in the field of subunit vaccines. First, our approach is a simple and efficient way to trigger robust CTL responses, whereas most other vaccine technologies fail to do so [4]. Second, very short MHC class I epitope can be used if a MHC class II epitope is embedded within the peptides, or if a TLR agonist is added to the vaccine formulation. Finally, very low doses of antigens are required. For example, 50 μg of the poorly immunogenic gp100 epitope was insufficient to trigger a CTL response when administered with the classic combination of IFA plus a TLR9 agonist [22]. By contrast, as little as 0.5 μg of the epitope was sufficient to trigger a significant CD8+ immune response when using our formulation. The advantages of our melanin-nanoparticle vaccine over other nanoparticle-vaccines such as liposomes or natural polymers are under study. However, the superior efficacy of our formulation when compared to the combination of IFA and a TLR9 agonist, which is one of the best adjuvant known to trigger CTL responses [22,23], suggests that our approach deserves further developments.

In conclusion, the conjugation of synthetic melanin to short peptides represents a very simple means of triggering T-cell response. This approach should be particularly useful for immunizations against cancer, for which immunizations against neo-epitopes are currently under intense investigations [1,2].

## Materials and methods

### Peptides and synthesis of melanin

The KVPRNQDWL (gp100), FSHHNIIRL (EphA2), SIINFEKL, **SQAVHAAHAEINEAGR,** SMLVLLPKKVSGLKQLESIINFEKLTKWTS (pOVA30) and SLK**ISQAVHAAHAEINEAGR**LRGSIINFEKLTKWR (pOVA35) endotoxin free peptides were purchased from Genosphere Biotechnologies (Paris, France). These peptides correspond to, or contain, H-2b epitopes (underlined) of the human glycoprotein 100 [20], the mouse Ephrin A2 [34] and the ovalbumin protein [35], respectively. The pOVA35 peptide also contains a mouse MHC class II epitope (bold) [36]. Stock solutions of peptides (10 mg/ml) and L-Dopa (2.2 mg/ml) (Sigma-Aldrich, Saint-Quentin-Fallavier, France) were prepared in Milli-Q purified water. For the preparation of synthetic melanin, peptides (10 μg/mouse, unless specified) were mixed with L-Dopa (100 μg/mouse, unless specified). The solutions were then incubated for 18 h at pH 8.5 under vigorous agitation (Eppendorf Thermomixer, 1000 rpm, 20°C) to ensure continuous oxygenation of the solution.

### Physico-chemical characterization

UV visible spectra were obtained using a JASCO V630 spectrophotometer (JASCO, Lisses, France). The solution of polymerizing L-Dopa was diluted 1/20, and spectra were recorded using 1cm path length quartz cuvette after different incubation times. Particle sizes were determined by transmission electron microscopy (Tecnai 12, ImagoSeine platform, France). Samples were prepared by depositing a drop of the above solution on carbon-coated copper grids placed on a filter paper. Fourier transform infrared (FTIR) spectra were recorded on a Tensor 27 spectrophotometer (Bruker, Karlsruhe, Germany) at a resolution of 1 cm^-1^, and the data treatment was performed using the Opus program. Because of interfering vibrations of H_2_O at 1645 cm^-1^, spectra were recorded in D_2_O. Deuteration experiments were performed by drying the samples and dissolving them in 1.5 μl D_2_O solution (>99.8% purity, Euriso-Top; CEA, Saclay, France). Solutions were deposited between two ZnSe windows.

Tricine-SDS-PAGE analysis was performed according to the protocol published by Schägger [37]. Briefly, samples were mixed with 5x sample buffer (containing glycerol, SDS, β-mercapto-ethanol and bromophenol blue) and heated for 4 min at 95°C. Aliquots containing 2 μg of peptide in a final volume of 12.5 μl were loaded on an electrophoresis gel using 4%, 10% and 16% acrylamide/Bis (29:1) for the stacking, spacer and resolving gel, respectively. Following electrophoresis, the gels were stained with Coomassie Brilliant Blue R-250 and imaged with the ChemiDoc XRS+ system (Bio-Rad Laboratories, Marnes la Coquette, France).

### Mice and immunization protocols

Female C57BL/6, or BALB/c mice (Janvier Labs, Le Genest-Saint-Isle, France) were 5–6 weeks old at the initiation of the experiment and were kept under specific-pathogen-free conditions. All animal experiments were approved by the ethics committee of Paris Descartes University (Project APAFIS #5337 N° 2016021517305775) and performed in accordance with European Union guidelines for animal experiments. Peptides (10 μg/mouse, unless specified) mixed with L-Dopa (100 μg/mouse, unless specified) were incubated for 18 h under the above described conditions. When specified, the phosphorothioate oligonucleotide CpG-28 (5’-TAAACGTTATAACGTTATGACGTCAT) (Oligovax, Paris, France), a B-type CpG-ODN [9, 38], or polyinosinic:polycytidylic acid (poly I:C), a TLR3 agonist (Sigma-Aldrich, Saint-Quentin-Fallavier, France) were added to the vaccine formulations (10 μg/mouse) just before the immunizations. Mice were immunized subcutaneously in the flank (100 μl/injection). As controls, some mice were injected with peptides + 50 μg of CpG-28 emulsified in incomplete Freund’s adjuvant (Sigma-Aldrich, Saint-Quentin-Fallavier, France). Mice were euthanized on the indicated day by cervical dislocation. Spleens and inguinal lymph nodes were surgically removed under aseptic conditions.

### Histological studies

The inguinal lymph nodes were surgically resected, fixed in 4% neutral buffered formalin for 24 h and embedded in paraffin. Five-micron-thick sections were then cut and stained with Fontana-Masson stain. The slides were reviewed by a pathologist (A.M.) using an Olympus BX51 light microscope, and images were obtained using a SPOT Insight Digital Camera (Diagnostic Instruments).

### T-cell responses

Epitope-specific IFNγ production by splenocytes was determined as previously described [9]. Briefly, single-cell suspensions of splenocytes (5 x 10^5^ cells/well) were stimulated at 37°C in 5% CO_2_ for 21 h with 10 μg/ml of the epitopes KVPRNQDWL for human gp100, FSHHNIIRL for murine EphA2, SIINFEKL or SQAVHAAHAEINEAGR for ovalbumin (Ovalbumine MHC class I and class-II epitopes, respectively). When spots indicating IFNγ production appeared, they were counted using a Cellular Technology Ltd system and analyzed using ImmunoSpot 5.0.3 software (Cellular Technology Ltd., Ohio, USA). The results are presented as the mean of triplicate wells and the number of IFNγ spot-forming cells (SFCs) per 5 x 10^5^ cells.

### Multiparametric flow cytometry and antibodies

Single-cell suspensions of splenocytes (1 x 10^6^ cells/well) were first stained for 45 min at 4°C with H-2Kb (SIINFEKL) dextramers conjugated to phycoerythrin (PE) (Immudex, Copenhagen, Denmark). Cells were then incubated with Pacific Blue^™^ anti-mouse CD3 (Biolegend, San Diego, CA, US), anti-mouse CD8a APC-eFluor^®^ 780 (eBioscience, San Diego, CA, US) and anti-mouse CD62L (L-Selectin) Alexa Fluor^®^ 700 (eBioscience) antibodies for 30 min at 4°C. After fixation and permeabilization using Fix/Perm buffer (eBioscience), cells were intracellularly stained with anti-human/mouse T-bet PE-Cyanine7 (eBioscience) and Alexa Fluor^®^ 647 anti-human/mouse Granzyme B (Biolegend) antibodies for 1 h at 4°C. Flow cytometry analysis was performed using a BD LSRII flow cytometer and BD FACSDIVA software (Becton Dickinson, NJ, US). The results were analyzed using FLOWJO software.

### Tumor models and *in vivo* CD8^+^ T-cell depletion

Under anesthesia with 2.5% isoflurane, C57BL/6 mice were injected subcutaneously with 5 x 10^4^ cells stably transfected with a plasmid expressing chicken ovalbumin (E.G7-OVA) (American Type Culture Collection, ATCC, USA). Mice were then immunized subcutaneously on days 4 and 18 after tumor graft. Tumors were measured every 3–4 days using calipers, and the tumor volumes were calculated using the following formula: π/6 x length x width^2^. Mice were sacrificed when tumors reached a maximal diameter of 20 mm. In the same tumor model, CD8^+^ T cells were depleted *in vivo* as follows: 100 μg of anti-CD8 mAbs (rat IgG2b mAb, clone YTS 169.4 from Proteogenix, France) per mouse or isotype control mAbs were injected intraperitoneally two days before therapeutic vaccination and then once per week until the end of the experiment.

### Statistics

For statistical analysis, continuous variables are presented as the mean for normally distributed variables and as the median for non-parametric variables. Associations between non-normally distributed variables were calculated using the Mann–Whitney test and between normally distributed variables by the paired Student t-test. Differences in tumor size among the various groups were determined using the ANOVA repeated-measures test. All reported P-values were based on two-sided tests at a significance level of 0.05. The Bonferonni’s correction was applied in case of multiple tests within the same experiment. All statistical analyses were performed using Statview 5.0 software^®^.

## Supporting information

S1 Fig CTL response after subcutaneous immunizations in C57BL/6 mice with gp100 + poly I:C or gp100-melanin + poly I:C (poly I:C = Polyinosinic:polycytidylic acid; 10μg/mouse). Mice were sacrificed 8 days after immunizations. (n = 8 mice/group with pooled data from 2 different experiments). SFC: spot-forming cells; Bars = median. *p<0.001.
(PPTX)

S2 Fig Evolution of the CTL response after 2 subcutaneous immunizations (day 0 and day 14) with gp100-melanin + CpG. Each point represents an individual mouse (n = 8 mice/group with pooled data from 2 different experiments of 4 mice each). SFC: spot-forming cells; Bars = median. * p < 0.01; **p<0.001, when compared to splenocytes stimulated with control epitopes.
(PPTX)
