# Supplementary figures and images for: Synthetic melanin bound to subunit vaccine antigens significantly enhances CD8+ T-cell responses
Source: PLoS One. 2017 Jul 17;12(7):e0181403. doi: 10.1371/journal.pone.0181403 (PMC5513539; doi:10.1371/journal.pone.0181403)

## Slide 1
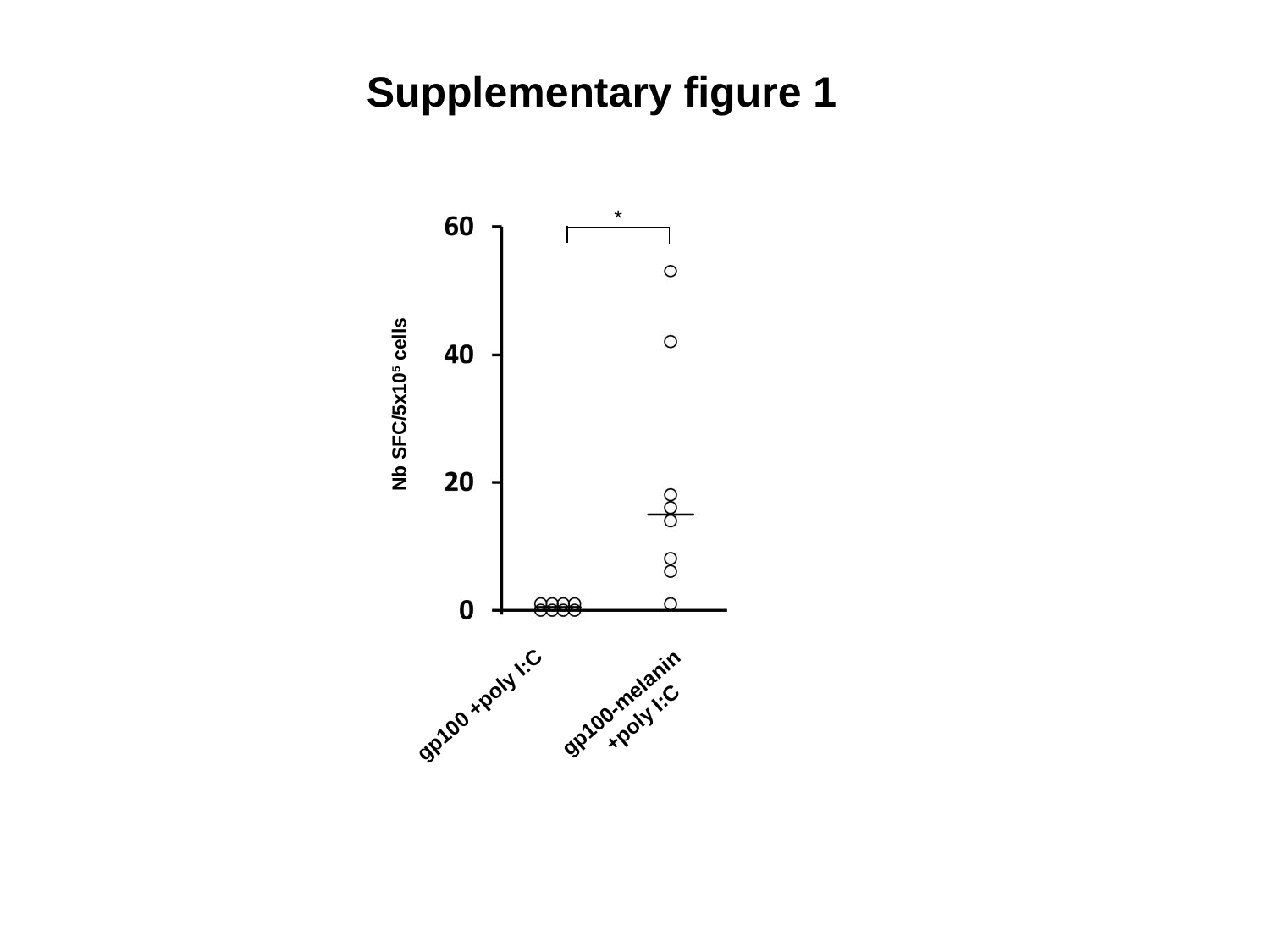

# Supplementary figure 1
*
Nb SFC/5x105 cells
gp100-melanin
+poly I:C
gp100 +poly I:C

Supplement: S1 Fig — Mice were sacrificed 8 days after immunizations. (n = 8 mice/group with pooled data from 2 different experiments). SFC: spot-forming cells; Bars = median. *p<0.001. (PPTX) [file pone.0181403.s001.pptx]

## Slide 1
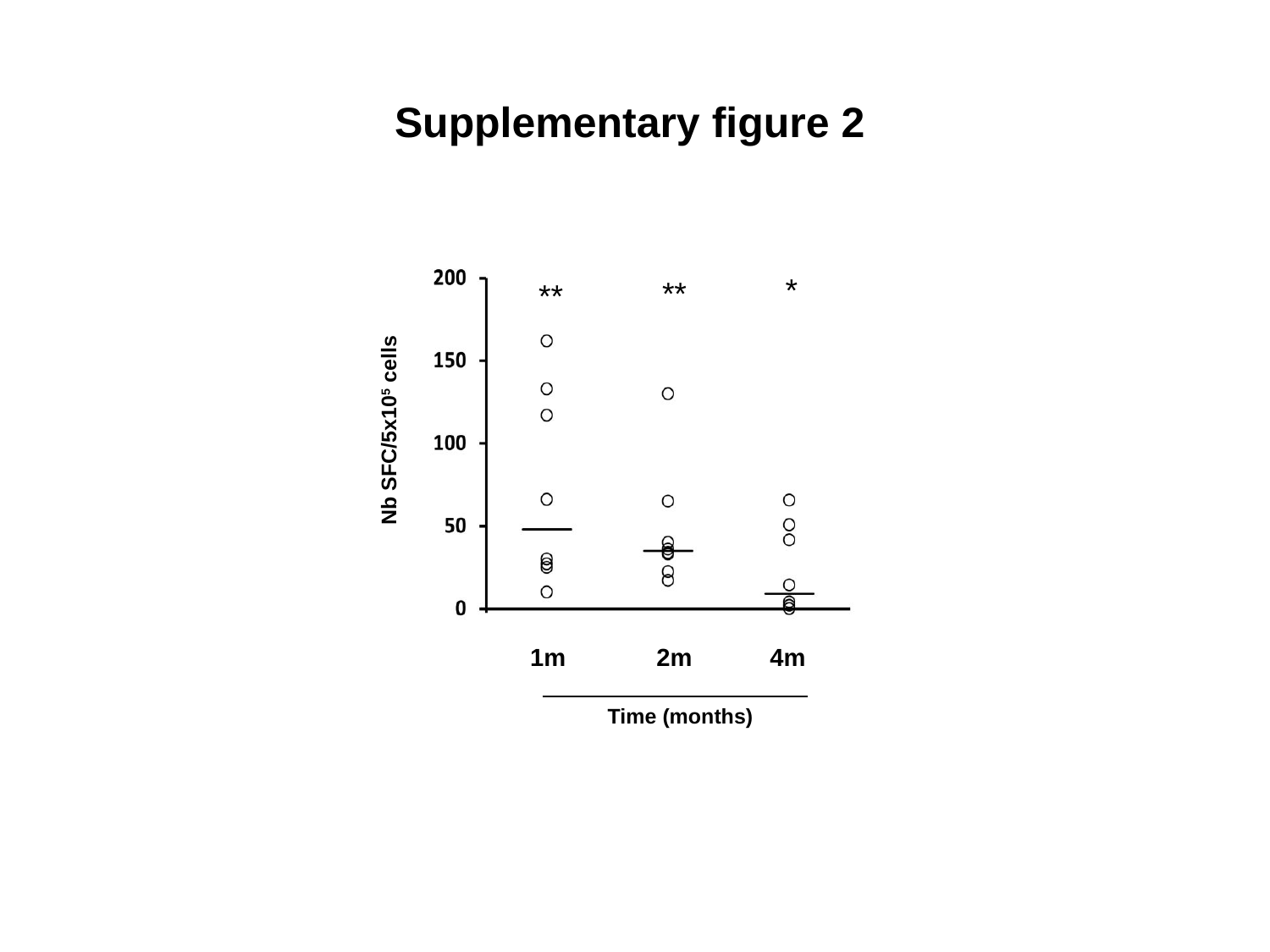

# Supplementary figure 2
*
**
**
Nb SFC/5x105 cells
1m
2m
4m
Time (months)

Supplement: S2 Fig — Each point represents an individual mouse (n = 8 mice/group with pooled data from 2 different experiments of 4 mice each). SFC: spot-forming cells; Bars = median. * p < 0.01; **p<0.001, when compared to splenocytes stimulated with control epitopes. (PPTX) [file pone.0181403.s002.pptx]
